# Low-level laser irradiation induces *in vitro* proliferation of mesenchymal stem cells

**DOI:** 10.1590/S1679-45082014AO2824

**Published:** 2014

**Authors:** Carlos Augusto Galvão Barboza, Fernanda Ginani, Diego Moura Soares, Águida Cristina Gomes Henriques, Roseana de Almeida Freitas

**Affiliations:** 1 Universidade Federal do Rio Grande do Norte, Natal, RN, Brasil.; 2 Universidade Federal de Pernambuco, Recife, PE, Brasil.

**Keywords:** Laser therapy, Cell proliferation, Stem cells, Bone marrow, Adipose tissue

## Abstract

**Objective:**

To evaluate the effect of low-level laser irradiation on the proliferation and possible nuclear morphological changes of mouse mesenchymal stem cells.

**Methods:**

Mesenchymal stem cells derived from bone marrow and adipose tissue were submitted to two applications (T0 and T48 hours) of low-level laser irradiation (660nm; doses of 0.5 and 1.0J/cm^2^). The trypan blue assay was used to evaluate cell viability, and growth curves were used to analyze proliferation at zero, 24, 48, and 72 hours. Nuclear alterations were evaluated by staining with DAPI (4'-6-diamidino-2-phenylindole) at 72 hours.

**Results:**

Bone marrow-derived mesenchymal stem cells responded to laser therapy in a dose-dependent manner. Higher cell growth was observed when the cells were irradiated with a dose of 1.0J/cm^2^, especially after 24 hours (p<0.01). Adipose-derived mesenchymal stem cells responded better to a dose of 1.0J/cm^2^, but higher cell proliferation was observed after 48 hours (p<0.05) and 72 hours (p<0.01). Neither nuclear alterations nor a significant change in cell viability was detected in the studied groups.

**Conclusion:**

Low-level laser irradiation stimulated the proliferation of mouse mesenchymal stem cells without causing nuclear alterations. The biostimulation of mesenchymal stem cells using laser therapy might be an important tool for regenerative therapy and tissue engineering.

## RESUMO

**Objetivo:**

Avaliar o efeito da terapia com laser de baixa intensidade sobre a proliferação e as possíveis alterações morfológicas nucleares em células-tronco mesenquimais de camundongos.

**Métodos:**

Célulastronco mesenquimais derivadas da medula óssea e do tecido adiposo foram submetidas a duas aplicações (T0 e T48 horas) de laser de baixa intensidade (660nm; doses de 0,5 e 1,0J/cm^2^). O ensaio de azul de tripan foi utilizado para a avaliação da viabilidade celular, e curvas de crescimento foram usadas para avaliar a proliferação das células em zero, 24, 48, e 72 horas. Alterações nucleares foram avaliadas por coloração com DAPI (4-6-diamidino-2-fenilindolo) em 72 horas.

**Resultados:**

As células-tronco mesenquimais derivadas da medula óssea responderam a terapia com laser de forma dose-dependente. Um maior crescimento celular foi observado quando as células foram irradiadas com dose de 1,0J/cm^2^, especialmente depois de 24 horas (p<0,01). As células-tronco mesenquimais derivadas do tecido adiposo responderam melhor à dose de 1,0J/cm^2^, com maior proliferação após 48 (p<0,05) e 72 horas (p<0,01). Nem alterações nucleares nem a mudança significativa na viabilidade celular foi detectada nos grupos estudados.

**Conclusão:**

Laser de baixa intensidade estimulou a proliferação de células-tronco mesenquimais sem causar alterações nucleares. A bioestimulação de células-tronco mesenquimais por laserterapia pode ser uma ferramenta importante para a terapia regenerativa e a engenharia tecidual.

## INTRODUCTION

Mesenchymal stem cells (MSCs) are undifferentiated cells with important potential for applications in cell therapy because of their capacity for self-renewal, proliferation, and differentiation into diverse types of specialized cells.^(1)^ Adult MSCs are responsible for the replacement of damaged tissues at the site where they reside. These cells can be isolated from various tissues, such as bone marrow,^(2)^ umbilical cord,^(3)^ dental pulp,^(4)^ periodontal ligament,^(5)^ and adipose tissue.^(6)^ MSCs derived from adipose tissue (ADSCs) have a differentiation potential similar to that of bone marrow-derived MSCs (BMSCs) and can differentiate into different cell types such as adipocytes, chondrocytes, osteoblasts, and myoblasts.^(7)^

Studies have shown that the higher the proliferation of MSCs, the greater the regenerative and healing capacity of the tissues where they reside. Within this context, low-level laser irradiation (LLLI) has been shown to be effective in a variety of medical conditions such as mucosal healing, skin ulcers, dermatitis, and mucositis by exerting positive biomodulatory effects on MSCs.^(8,9)^ This capacity of accelerating the healing process is most likely related to the finding that LLLI promotes cell proliferation. However, the underlying molecular mechanisms for this process are still not completely understood.^(10)^ It has been suggested that the energy of the laser is absorbed by intracellular chromophores and converted into metabolic energy, which is then used by the mitochondrial respiratory chain to produce ATP and increasing DNA activity and the synthesis of RNA and proteins.^(11,12)^

The visible, infrared or ultraviolet spectra of light can be used for LLLI. According to the literature, the visible light spectrum ranging from 600 to 700nm^(13)^ yields the most effective results in terms of cell proliferation and differentiation. Yu et al.^(14)^ reported higher production of fibroblast growth factor when fibroblasts were irradiated with a 660-nm laser beam. Pourreau-Schneider et al.^(15)^ showed that laser irradiation at a wavelength of 632.8nm induced the transformation of fibroblasts into myofibroblasts in culture. Furthermore, laser light of 632.8nm was found to increase cell proliferation in keratinocyte cultures^(16)^ and to stimulate the secretion of cytokines IL-1 and IL-8.^(17)^ Stein et al.^(18,19)^ observed higher proliferation, differentiation, and maturation of osteoblasts when respectively irradiated at 632.8 and 670nm.

LLLI has been shown to stimulate the growth, proliferation and differentiation of different types of cells in culture, including keratinocytes, fibroblasts, endothelial cells, myoblasts, and osteoblasts, by exerting positive biomodulatory effects.^(19,20)^ However, little is known about the effect of laser therapy on MSCs.^(4,12,21)^ Because the proliferation of MSCs is usually slow and the yield of these cells after first harvest is low, a therapeutic tool that increases their proliferation without causing molecular damage while maintaining their specific characteristics is important for effective clinical application of these cells.

## OBJECTIVE

To evaluate the cell yield and DNA damage in bone marrow-derived mesenchymal stem cells and adipose tissue-derived mesenchymal stem cells treated with two doses (0.5 and 1.0J/cm^2^) of LLLI at 660nm.

## METHODS

The study was approved the Animal Ethics Committee of *Universidade Federal do Rio Grande do Norte*, Brazil (protocol 008/2009), and performed at the Department of Morphology/Center of Biosciences of the institution, from 2010 to 2011.

### Cell culture

BMSCs and ADSCs were obtained from two 3-monthold male albino Swiss mice. BMSCs were extracted according to the protocol of Soleimani and Nadri.^(22)^ Briefly, femurs and tibias were dissected, and the bone marrow cavities were washed with α-MEM medium supplemented with 10% fetal bovine serum (FBS; Gibco, USA), 50mg/L gentamicin sulfate, and 2mg/L amphotericin (both from Cultilab, Brazil). For the isolation of ADSCs, an adipose tissue fragment was removed from the inguinal region of the animal and washed three times with α-MEM supplemented with antibiotics and antifungal agents (Gibco, USA). Next, the tissue fragment was digested enzymatically with collagenase (3mg/mL; Gibco, USA) for 1 h at 37°C.

The two cell types were cultured in plates containing α-MEM medium supplemented with 10% FBS in a humid atmosphere with 5% CO_2_ at 37°C. The culture medium was changed at intervals of 3 to 4 days until the cells reached 80 to 95% confluency.

**Figure 1:**
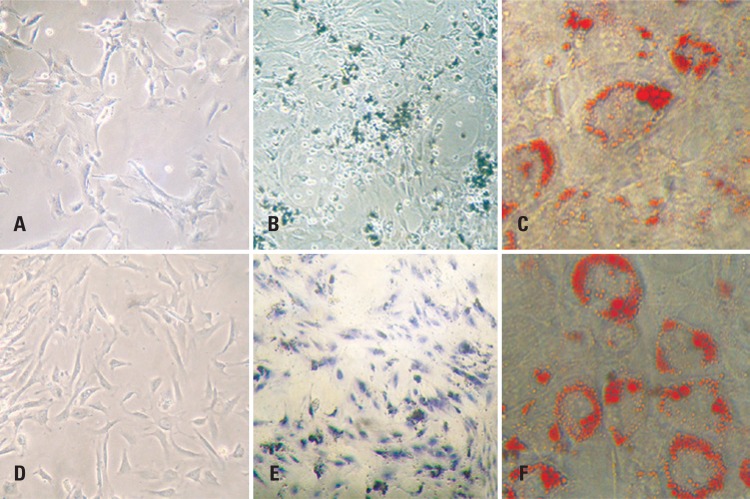
Photomicrograph of bone marrow (A to C) and adipose tissue (D to F) mesenchymal stem cells in the undifferentiated stage (A and D) and subjected to osteogenic (B and E) and adipogenic differentiation (C and F). Light microscopy, A and D: 40x; B and E: von Kossa stain, 40x; C and F: Oil Red stain, x100

To analyze the multipotent nature of the cells before laser therapy, BMSCs and ADSCs were cultured in osteogenic and adipogenic differentiation medium (StemPro^®^ Differentiation kits, Invitrogen Corp., Carlsbad, CA, USA) for up to 21 days. Analysis of the cells after this period using light microscopy revealed the characteristic morphology of osteoblastic and adipose cells (Figure 1).

### Laser irradiation

Third-passage (P3) cell cultures were irradiated with an InGaAlP diode laser (Kondortech Bio Wave LLLT Dual, Brazil) in the continuous mode using a power of 30mW, wavelength of 660nm, and doses of 0.5 and 1.0J/cm^2^. Each well treated with the 0.5 and 1.0J/cm^2^ doses was irradiated for 16 and 33 seconds, respectively, at zero and 48 hours, and the cells were analyzed at zero, 24, 48, and 72 hours after the first laser irradiation. For laser irradiation, the probe was directed perpendicular to each plate at a distance of 0.5cm from the cells. The cells were plated in such a way that was one well, between the seeded wells, was left empty to prevent the unintentional dispersion of light between wells during laser irradiation.

### Analysis of the effect of laser irradiation on cell proliferation and viability

The trypan blue assay was used to evaluate the cell viability during the experiment, and the obtained growth curves were used to establish the proliferation of cells that were submitted to laser therapy. The cells were cultured in 24-well plates at a density of 3x10^4^ cells/well, and trypan blue-stained cells were counted in a Neubauer chamber at zero, 24, 48, and 72 hours.

### Analysis of the effect of laser irradiation on cell nuclei

To evaluate nuclear morphological changes, the cells were stained with DAPI (4'-6-diamidino-2-phenylindole) to identify the presence of pyknotic nuclei and nuclear fragmentation after 72 hours.

### Statistical analysis

The results were submitted to nonparametric analysis. Differences between groups at each time point (zero, 24, 48, and 72 hours) were analyzed using the Kruskal-Wallis and Mann-Whitney tests. A level of significance of 5% was adopted (p<0.05).

## RESULTS

### Bone marrow-derived mesenchymal stem cells

**Figure 2:**
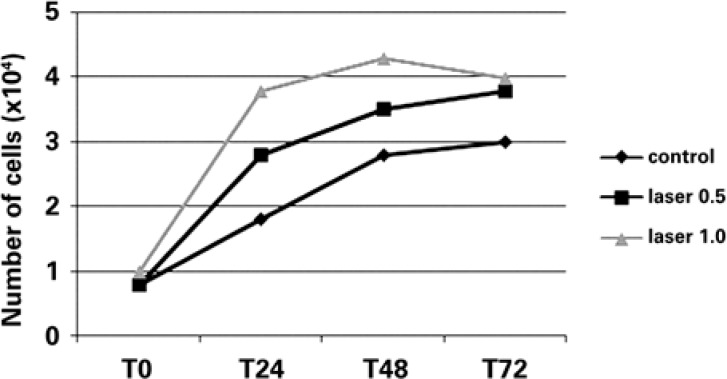
Growth curve of bone marrow-derived mesenchymal stem cells submitted or not submitted to laser irradiation over time

Figure 2 illustrates the growth curve of BMSCs in the different groups. The lowest proliferation was observed for the control group (no laser treatment) at all studied time points when compared to the irradiated groups.

Comparison of the two laser doses showed a tendency towards higher cell proliferation in the group treated with 1.0J/cm^2^, but this difference was only significant after 24 hours (Table 1). A significant difference in cell proliferation was observed between the control group and the groups treated with the two doses (0.5 and 1.0J/cm^2^) after 24 hours (Table 1), with both doses stimulating cell growth. No significant difference in cell viability was observed (Table 1) using the trypan blue exclusion method.

**Table 1:** Growth and viability of bone marrow-derived mesenchymal stem cells submitted or not submitted to laser irradiation at the different studied time points

	Control	Laser 0.5	Laser 1.0	p value*		
				Control *versus* 0.5	Control *versus* 1.0	0.5 *versus* 1.0
T0	0.8±0.3 100%	0.8±0.3 100%	1.0±0.0 93.4%	0.9266	0.0705	0.0705
T24	1.8±0.3 100%	2.8±0.3 94.9%	3.8±0.3 96.8%	0.0039**	0.0039**	0.0039**
T48	2.8±0.3 100%	3.5±0.0 97.8%	4.3±0.8 98.5%	0.0024**	0.0039**	0.0705
T72	3.0±0.0 98.6%	3.8±0.3 100%	4.0±0.0 100%	0.0024**	0.0013**	0.0705

Values of cell growth are reported as mean±standard deviation. Viability is reported as the percentage of viable cells. No significant change in cell viability was observed.

* Mann-Whitney test;

** significant differences (p<0.01).

### Adipose-derived mesenchymal stem cells

**Figure 3:**
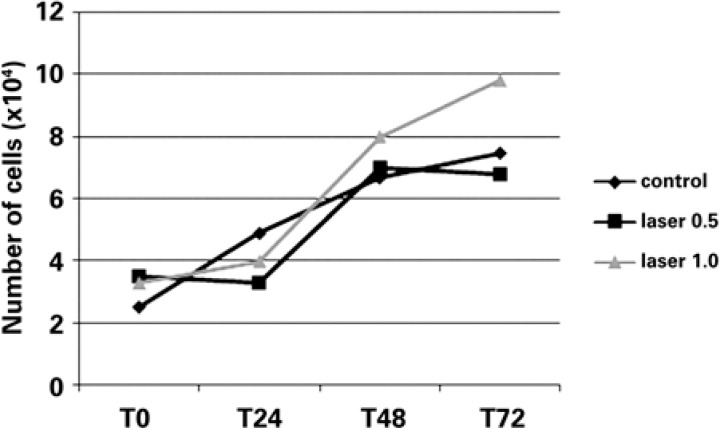
Growth curve of adipose-derived mesenchymal stem cells submitted or not submitted to laser irradiation over time

The proliferation of ADSCs tended to increase in all studied groups (Figure 3). A tendency towards higher proliferation was observed in the group irradiated with the dose of 1.0J/cm^2^ after 24 hours when compared with the control group and the group irradiated with 0.5J/cm^2^.

Comparison of the cell proliferation of the laser-irradiated groups showed a significant difference only after 72 hours (Table 2). Laser irradiation using a dose of 1.0J/cm^2^ seemed to positively influence the proliferation of ADSCs, and a distinct behavior was observed over time when the mean cell proliferation was compared between the laser-irradiated groups and the control group (Table 2). The trypan blue exclusion assay showed no difference in cell viability in the studied groups.

**Table 2:** Growth and viability of adipose-derived mesenchymal stem cells submitted or not submitted to laser irradiation at the different studied time points

	Control	Laser 0.5	Laser 1.0	p value*		
				Control *versus* 0.5	Control *versus* 1.0	0.5 *versus* 1.0
T0	2.5±0.5 100%	3.5±0.5 100%	3.3±0.3 93.8%	0.0239**	0.0239**	0.4871
T24	4.9±0.9 98.1%	3.3±0.3 94.4%	4.0±1.1 100%	0.0041**	0.3674	0.4871
T48	6.7±0.6 98%	7.0±1.6 95.9%	8.0±1.1 100%	0.8671	0.0383**	0.1613
T72	7.5±0.8 96.8%	6.8±0.8 96.9%	9.8±1.4 100%	0.1659	0.0084**	0.0039**

Values of cell growth are reported as mean±standard deviation. Viability is reported as the percentage of viable cells. No significant change in cell viability was observed.

* Mann-Whitney test;

** significant differences (p<0.05).

### Effect of laser irradiation on cell nuclei

**Figure 4:**
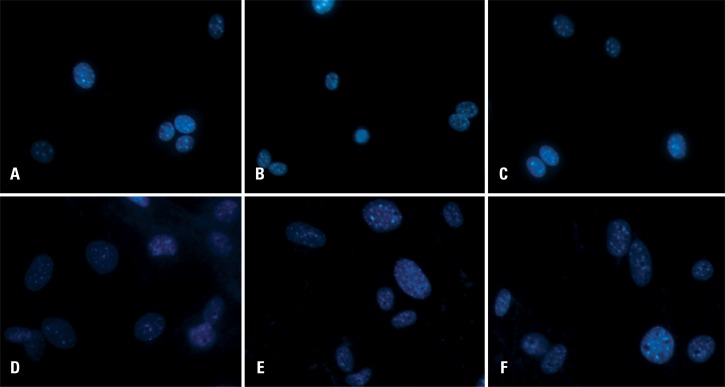
Micrograph of bone marrow-derived mesenchymal stem cells (A to C) and adipose tissue-derived mesenchymal stem cells (D to F) stained with DAPI, showing no nuclear morphological changes. (A and D) Control; (B and E) laser 0.5J/cm^2^ and (C and F) laser 1.0J/cm^2^

No nuclear change (nuclear fragmentation or pyknotic nuclei) was detected in BMSCs or ADSCs (Figure 4).

## DISCUSSION

LLLI is used to stimulate the proliferation and differentiation of different types of cells.^(4,6,9,11,23)^ However, biostimulation is not always observed because a variety of factors influence this process.^(24)^ In this respect, *in vitro* biostimulation depends on laser-related parameters such as wavelength, dose, power and time of irradiation,^(11,23,25)^ type of cell irradiated,^(26)^ and the physiological characteristics of the cells at the time of irradiation.^(23,25)^ As a consequence of these factors, the interaction of laser light with cells and tissues can stimulate or inhibit cell proliferation.

Pinheiro et al.^(27)^ recommended lower doses for the irradiation of mucosa and skin wounds because the absorption and spreading of light are greater due to the lack of an optical barrier. According to the authors, *in vitro* studies normally reflect the same conditions as observed in open wounds, i.e., the lack of an optical barrier, and lower doses are, thus, indicated in the case of biostimulation. On the basis of this hypothesis, doses of 0.5 and 1.0J/cm^2^ were used in the present study because the objective was to stimulate the proliferation of BMSCs and ADSCs. According to Karu,^(28)^ a dose increase damages photoreceptors, which reduces the biomodulatory effect of the laser as a result of the inhibition of metabolism and consequent cell death. Kreisler et al.^(11)^ supported this hypothesis by demonstrating that laser irradiation using a dose of 4.0J/cm^2^ exerted a stimulatory effect on cell proliferation, whereas high doses seemed to exert a negative effect on this biological process.

Studies investigating the effects of laser irradiation on stem cells have shown that this therapy can increase cell proliferation^(18,21)^ and contributes to the differentiation of these cells.^(18,29)^ Wavelengths of 600 to 700nm were used when the objective was to stimulate cell proliferation and differentiation.^(13)^

One study demonstrated that irradiation of human ADSCs using a laser at a wavelength of 635nm and dose of 5.0J/cm^2^ positively influenced cell proliferation and viability, as well as the expression of proteins, such as epidermal growth factor.^(6)^ These results agree with the present findings that show an increased number of cells. However, lower doses were used (0.5 and 1.0J/cm^2^) that elicited responses similar to those reported in the literature. We believe that lower doses reduce the risk of cell damage and promote the proliferation of stem cells, which keeps their initial characteristics intact. In addition, laser therapy showed a dose-dependent effect in the present study, as indicated by the higher proliferation rate of BMSCs and ADSCs when irradiated with a dose of 1.0J/cm^2^ compared with 0.5J/cm^2^. Stein et al.^(19)^ observed that LLLI using a wavelength of 670nm and doses of 1.0 and 2.0J/cm^2^ exerted a positive biomodulatory effect on the growth and differentiation of human osteoblasts during the first 72 hours after irradiation. The best results were obtained with the dose of 1.0J/cm^2^, which was in agreement with the present findings showing higher proliferation of BMSCs and ADSCs treated with a dose of 1.0J/cm^2^ over the same period of time (72 hours).

Moore et al.^(26)^ studied the effect of laser irradiation at different wavelengths (625, 635, 645, 655, 665, 675, and 810nm) on two types of cells (fibroblasts and endothelial cells) by evaluating their cell proliferation rates after 72 hours of culture. The authors found an increase in the growth of endothelial cells at all tested wavelengths. The same was observed for fibroblasts, except for the wavelength of 810nm. Similar results regarding the proliferation of fibroblasts have been reported by Evans and Abrahamse,^(30)^ who evaluated wavelengths of 632.8 and 830nm. The authors observed more effective stimulation of fibroblast proliferation for cells treated with the 632.8nm laser, suggesting that the operating wavelength of the laser interfered with the cell response. The wavelength used in the present study (660nm) was within the range that was employed in biostimulation studies, and, as reported in the literature, we observed a positive effect on the proliferation of the cells tested.

Bouvet-Gerbettaz et al.^(9)^ analyzed the influence of LLLI on the proliferation and differentiation of BMSCs in suspension and observed the presence of colonyforming units of average size (diameter <0.5mm) in the control and laser-treated groups after 7 days, with no significant difference between the groups. These results disagree with the present study in which significant differences in the proliferation rate of BMSCs and ADSCs were observed between the irradiated and control groups. Factors that might have influenced the lack of a difference in the study of Bouvet-Gerbettaz et al.^(9)^ include the wavelength (808nm), dose used (4.0J/cm^2^), and the study design. As discussed earlier, wavelengths outside the range of 600 to 700nm and high irradiation doses may exert inhibitory effects on cell proliferation.^(13,23,25)^

In the present study, BMSCs responded to laser therapy in a dose-dependent manner, and a similar cell proliferation curve at the two doses tested (0.5 and 1.0J/cm^2^) and a cumulative action of these doses over time was observed. However, a higher proliferation rate was observed for cells irradiated with 1.0J/cm^2^, especially after 24 hours of culture. ADSCs responded better to the irradiation dose of 1.0J/cm^2^, with higher cell proliferation being observed after 48 and 72 hours of culture. These results agree with the suggestion of some investigators that the action of the laser is directly related to the irradiation dose, physiological state of the cell, cell line, and wavelength used.^(20,23)^

The cell line that is used is a factor that influences the effect of laser therapy. In this respect, de Villiers et al.^(7)^ evaluated the effect of irradiation using a 636nm diode laser at a dose of 5.0J/cm^2^ on the proliferation of human ADSCs. The authors used one cell line that was obtained during surgery and a commercial cell line and obtained distinct results. No significant difference in proliferation was observed for the commercial line at any of the studied time points (24, 48, and 72 hours). In contrast, significant differences in cell proliferation were observed at 24 and 72 hours post-irradiation between cells obtained during surgery and the nonirradiated control. Hou et al.^(29)^ used LLLI to increase the proliferation rate of BMSCs by applying irradiation doses of 0.5, 1.0, 2.0 and 5.0J/cm^2^ with a 635nm laser; and the authors found no significant differences in cytotoxicity between the non-irradiated and irradiated groups. However, irradiated BMSCs presented a significantly higher proliferation rate than control cells. The dose of 0.5J/cm^2^ was found to be the most effective; however, the time of evaluation was longer in that study. Nevertheless, these results agree with the present study in which the cell yield was significantly higher for irradiated BMSCs when compared to the control group.

Tuby et al.^(21)^ demonstrated that laser therapy promotes the proliferation of MSCs and cardiac stem cells *in vitro.* In this study, the cells were irradiated with an 804-nm diode laser (AsGa) at doses of 1.0 to 3.0J/cm^2^, and proliferation of the two cell types was significantly increased after laser irradiation when compared with the non-irradiated control group. The utilized doses induced no adverse effect on the cells, which was also observed in the present study in which no alterations compatible with DNA damage, such as pyknotic or fragmented nuclei, were detected in the cells submitted to laser therapy. However, other alterations such as chromosomes deletions or translocations could not be excluded by this method; therefore, karyotyping is essential for further studies.

## CONCLUSION

The present study showed that low-level laser irradiation promoted the proliferation of bone marrow-derived mesenchymal stem cells and adipose tissue-derived mesenchymal stem cells. Because stem cells isolated from different sources usually present a low yield and low proliferation rate, low-level laser irradiation may be a useful tool for tissue engineering using stem cells. In this respect, laser therapy permits a significant increase in the initial number of stem cells before differentiation, thus increasing the number of differentiated cells for tissue engineering and regenerative and healing processes. However, further studies are needed to standardize the laser parameters and to test other cell types to improve the yield of cells in culture.
